# Metabolomic biomarkers discovery across chronic gastritis to gastric cancer progression

**DOI:** 10.1038/s41598-025-19005-7

**Published:** 2025-09-29

**Authors:** Le Yang, Jie Wang, Peng Li, Yuxi Guo, Siyuan Li, Shuangyu Liu, Yingying Lou, Jinlong Qi, Qian Yang

**Affiliations:** 1 Department of Pharmacology, Hebei Medical University, Shijiazhuang, 050017 China; 2 Collaborative Innovation Center of Hebei Province for Mechanism, Diagnosis and Treatment of Neuropsychiatric Diseases, Shijiazhuang, 050017 China; 3 The Key Laboratory of Neural and Vascular Biology, Ministry of Education, Shijiazhuang, 050017 China; 4 Hebei Provincial Hospital of Chinese Medicine, Shijiazhuang, 050011 China; 5 Qinhuangdao Hospital of Traditional Chinese Medicine, Qinhuangdao, 066000 China; 6 Key Laboratory of Turbidity Toxin Syndrome (Hebei), Shijiazhuang, 050013 China

**Keywords:** Gastric cancer, Metabolomics, Dehydroepiandrosterone sulfate, L-threonic acid, Biomarker, Gastrointestinal cancer, Tumour biomarkers, Diagnostic markers

## Abstract

Gastric cancer (GC) is a severe malignancy characterized by late diagnosis, poor prognosis, and low survival rates. Its progression is often linked to chronic non-atrophic gastritis (CNAG) and chronic atrophic gastritis (CAG), which show atypical symptoms. Identifying biomarkers for CNAG, CAG, and GC progression is crucial for earlier diagnosis and prevention. This study conducted non-targeted metabolomics on 81 clinical samples (17 controls; 23, 23, and 18 from CNAG, CAG, and GC patients, respectively) using ultra-high-performance Liquid chromatography and high-resolution mass spectrometry. A total of 763 metabolites were identified, of which eight metabolic pathways were in dysregulation at different disease stages. Disease progression showed pronounced disruptions in amino acid, Lipid, and microbial metabolism. Targeted metabolomics identified 56 metabolites, with significant differences in O-(4,8-dimethylnonanoyl) carnitine and dehydroepiandrosterone sulfate (DHEAS). DHEAS and L-threonic acid (L-TA) were validated as biomarkers, with detection methods developed and applied to confirm their clinical significance. These findings enhance understanding of CNAG, CAG, and GC progression and provide validated biomarkers for potential clinical application in GC diagnosis and treatment.

**Supplementary Information:**

The online version contains supplementary material available at 10.1038/s41598-025-19005-7.

## Introduction

Gastric cancer (GC) is one of the most prevalent types of solid tumor of the digestive system and is the third leading cause of cancer-related mortality worldwide^1–3^. Recent findings from the National Cancer Center of China in 2022 have revealed that GC has become the fourth most prevalent malignant tumor in China and is now the third leading cause of cancer-related death^4^. Owing to the absence of distinctive early symptoms and the difficulty in differentiating GC from common gastritis^3^a majority of patients are diagnosed when their disease is advanced and the best opportunity for treatment has been missed, resulting in a median overall survival of only 8 months^5^^[,6^;. Therefore, there are formidable challenges associated with this disease, including overcoming the risks of missing the window for surgical intervention and resistance to chemotherapy, which contribute to the poor prognosis.

According to the histopathology of the gastric mucosa, chronic gastritis is commonly categorized as chronic non-atrophic gastritis (CNAG), chronic atrophic gastritis (CAG), or other special types^7–9^. CNAG, which is most frequently encountered, is characterized by inflammation of the superficial gastric mucosa, is associated with symptoms such as eructation, upper abdominal pain, acid reflux, and diminished appetite, and is also closely associated with factors such as *Helicobacter pylori* infection, smoking, and alcohol consumption^10^. CAG represents a precursor of GC, but has nonspecific clinical manifestations^11^^[,12^;. Atrophy of the gastric mucosa is a pathologic change that predisposes toward carcinogenesis: there is a 10% probability of progression from CAG to GC, and this is a pivotal stage in the process of transformation from inflammation to carcinoma^12^^[,13^;. According to the widely accepted Correa cascade, the following pathologies develop in the following sequence: CNAG, CAG, intestinal metaplasia, intraepithelial neoplasia, and ultimately early GC^14–16^. The current gold-standard method for the diagnosis of CNAG, CAG, and GC is imaging (endoscopy), accompanied by the measurement of pathological indices, but these procedures are invasive and associated with poor patient compliance. Thus, there is an urgent need for the development of effective and convenient methodologies for the accurate clinical diagnosis of gastric ailments, thereby enabling the timely diagnosis and treatment of GC.

Metabolomics is a powerful means of characterizing biological systems. It has the capacity to amplify subtle differences in the genome and proteome that are reflected in the biochemical phenotype of organisms^17–19^. Metabolomic samples, which are predominantly derived from blood and urine, are readily accessible, and therefore the analyses are associated with high patient compliance. However, recent literature reviews have highlighted the absence of well-established biomarkers of the progression of CNAG, CAG, and GC^20–22^. Consequently, it is important to identify novel circulating biomarkers that would aid the diagnostic process, and in particular to facilitate the early diagnosis of the disease, thereby increasing the likelihood of successful treatment and improving the survival rate of patients with GC. A better understanding of the changes in molecular pathways that accompany the progression from CNAG to CAG and GC and the identification of pertinent biomarkers would not only permit a swifter diagnosis and improve the prognosis of patients, but would also facilitate the identification of novel drug targets, thereby guiding new drug development.

Mass spectrometry is an excellent analytic platform for metabolomic analysis, providing high sensitivity, reproducibility, and versatility. The analysis of samples containing a complex mixture of metabolites using ultra-high-performance liquid chromatography coupled with mass spectrometry (UPLC-MS) has been successfully used as a means of high-throughput metabolomics^23–25^. In the present study, we collected serum samples from individuals with CNAG, CAG, or GC, and healthy controls, and analyzed these using both untargeted and targeted metabolomics by means of UPLC-MS. This generated metabolomic profiles for GC and precancerous lesions, and we successfully identified and validated potential biomarkers for the clinical diagnosis and treatment of these conditions.

## Materials and methods

### Participants and sample collection

Serum samples were collected from the participants as part of a National Administration of Traditional Chinese Medicine Science and Technology Project. Between March and December 2022, 23 patients with chronic non-atrophic gastritis (CNAG group, *n* = 23, 12 men, 11 women), 23 with chronic atrophic gastritis (CAG group, *n* = 23, 11 men, 12 women), and 18 with gastric cancer (GC group, *n* = 18, 13 men, 5 women) were enrolled at Hebei Provincial Hospital of Traditional Chinese Medicine. In addition, 17 healthy individuals were recruited (10 men, 7 women) as a normal control group for both the non-targeted and targeted metabolomics studies. During July and August 2023, a total of 36 clinical samples were collected, comprising 9 samples from the Control group (4 men, 5 women), 9 from the CNAG group (6 men, 3 women), 9 from the CAG group (3 men, 6 women), and 9 from the GC group (6 men, 3 women), for the validation of the newly identified biomarkers. The demographic characteristics of the study subjects are presented in Table S1 and Table S2.

Ethics approval for the study was obtained from the Medical Ethics Review Board of Hebei Provincial Hospital of Traditional Chinese Medicine, and all the participants provided their informed consent. Rigorous participant selection criteria were used. The inclusion criteria for the healthy control group were as follows: availability of blood samples collected at the time of health examinations; absence of infections, such as Helicobacter pylori infection, and other diseases affecting gastric function; and no use of supplements or medications affecting gastric function testing during the month prior to sampling. The exclusion criteria for the disease groups were as follows: non-compliance with blood collection; menstruation, pregnancy, or lactation; metabolic disease; other diseases of the digestive system; and other factors that could have affected gastric function, such as diet and lifestyle. Blood samples were collected from patients in the fasting state, and the gold-standard method was used for the diagnosis of disease: pathologic confirmation using biopsy samples obtained during gastroscopy.

## Reagents and equipment

Dehydroepiandrosterone sulfate (DHEAS) sodium and L-threonic acid (L-TA) hemicalcium salt were purchased from Aladdin (Shanghai, China), and dexamethasone was purchased from Solario (Beijing, China). HPLC-grade acetonitrile and ammonium acetate were purchased from Fisher Scientific (United States).

For the non-targeted metabolomics study, an Ultimate 3000 ultra-high-performance liquid chromatography (UPLC) system was employed in conjunction with a Q Exactive Orbitrap high-resolution mass spectrometer (HRMS) (THERMO Fisher Scientific, Waltham, MA, USA). For the targeted metabolomics study and the quantitative analysis of biomarkers, an LC-30 A UPLC system (Shimadzu, Kyoto, Japan) was coupled to an API 4000 Q-Trap low-resolution mass spectrometer (LRMS) (SCIEX, Framingham, MA, USA).

## Sample processing

### Processing of metabolomics samples

Serum samples collected from the Control, CNAG, CAG, and GC groups were pooled in equal volumes to prepare quality control (QC) samples. One hundred-microliter samples of serum were aspirated, mixed with 300-µl acetonitrile, vigorously vortexed for 1 min, then centrifuged at 14,000 rpm for 10 min at 4 °C. A 100-µl aliquot of each supernatant was transferred to an injection vial, and 3-µl volumes were analyzed.

### Processing of candidate biomarker samples

The serum samples were stored at − 40℃. For each 100-µl serum or simulated serum sample, including QC and standard curve samples diluted using simulated blank serum, three volumes of acetonitrile solution containing 100 ng/ml dexamethasone (IS) was added. The mixture was vortexed, centrifuged at 14,000 rpm for 10 min at 4℃, and 100-µl aliquots of the supernatants were transferred to injection vials, then 5-µl aliquots were injected for analysis.

## LC-MS

### LC-MS conditions for non-targeted metabolomics

A Phenomenex HILIC chromatographic column (2.6 μm, 3.0 mm internal diameter × 100 mm) was employed, using acetonitrile as mobile phase (A) In positive mode, mobile phase B consisted of a solution of 10 mM ammonium acetate in 0.1% formic acid, and in negative mode, mobile phase B was a solution of 10 mM ammonium acetate. The gradient elution was performed as follows: 0–1 min 2% B, 1–16 min 2–50% B, 16–18 min 50% B, 18–18.5 min 50–2% B, 18.5–21 min 2% (B) The column temperature was 40℃ and the flow rate was 0.4 ml/min. For MS, we used an electrospray ionization source (ESI) with a spray voltage of 3.5 kV/3.2 kV (+/−), an ion source temperature of 350℃, and a capillary temperature of 320℃. The sheath gas flow rate was 40 arb and the auxiliary gas flow rate was 10 arb. Scanning was conducted separately for positive and negative ions using Full scan/ddMS2 mode, covering a mass range of 100 to 1,000 m/z. The scan resolution of the primary mass spectrum (MS1) and the secondary mass spectrum (MS2) were 70,000 FWHM and 17,500 FWHM, respectively. The collision energies for the secondary scan were set at 20, 40, and 60 eV, with an S-lens value of 60.

### LC-MS conditions for targeted metabolomics

The chromatographic conditions described above were used, and mass spectrometry was performed using an ESI source. The detection method was multiple reaction monitoring (MRM) in both positive and negative ion modes. The electrospray voltage was set at ± 4,500 V, curtain gas was used at 137.89 kPa, nebulizer gas was used at 310.26 kPa, auxiliary gas was used at 310.26 kPa, and the ion source temperature was 550℃. The declustering potentials were 60, 120, and 180 V for positive ion mode, and − 60, −120, and − 180 V for negative ion mode.

### Conditions for the simultaneous determination of potential biomarker concentrations using chromatography–mass spectrometry

The analysis column used was a Kinetex XB-C18 (2.6 μm, 3.0 mm internal diameter × 100 mm), and the mobile phases were 5 mmol/L ammonium acetate in water (phase B) and acetonitrile (phase A), with gradient elution (0–0.5 min 15–95% A; 0.5–4 min 95% A). The column temperature was 40℃ and the flow rate was 0.4 ml/min. Mass spectrometry detection was performed in MRM mode with negative ion monitoring, with an ESI ion source spray voltage at − 4,500 V, a curtain gas pressure of 20 psi, a nebulizer gas pressure of 45 psi, an auxiliary gas pressure of 45 psi, and an ion source temperature of 550℃. The selected ion pairs were m/z 135.2→75.1 (L-TA), m/z 367.2→97.1 (DHEAS), and m/z 391.3→361.3 (dexamethasone, IS).

### Development of an LC–MS method for the simultaneous determination of biomarker concentrations

We also developed a UPLC–MS/MS method for the simultaneous determination of L-TA and DHEAS in human serum. Bovine serum albumin powder dissolved in physiological saline was used to simulate blank human serum, and dexamethasone served as the IS. The established method underwent comprehensive methodologic validation in accordance with the guidelines of the China Food and Drug Administration (CFDA) and the U.S. Food and Drug Administration (FDA).

For the preparation of the L-TA standard curve, precise Weighing of the L-TA standard was performed, followed by dissolution in distilled water to create a stock solution with a final concentration of 1 mg/ml. The stock solution was then incrementally diluted with simulated blank serum to achieve final concentrations of 500, 1,000, 2,000, 5,000, 10,000, and 20,000 ng/ml, with which an L-TA standard curve was constructed. QC samples with concentrations of 600, 3,000, and 15,000 ng/ml were prepared. The standard DHEAS was precisely weighed, then dissolved in a small amount of DMSO, which was then diluted with acetonitrile/water (v/v = 1:1) to produce a DHEAS stock solution with a final concentration of 1 mg/ml. The stock solution was then stepwise diluted with simulated blank serum to achieve final concentrations of 50, 100, 200, 500, 1,000, and 2,000 ng/ml, with which a DHEAS standard curve was constructed. QC samples were prepared at concentrations of 80, 400, and 1,600 ng/ml.

### Data processing and statistical analysis

Non-targeted metabolomics data were preprocessed by Compound Discoverer 3.2.0.421 (Thermo Fisher Scientific), including peak alignment, signal correction, and extraction of secondary mass spectrometry ions. Metabolites were annotated using databases such as mzCloud, mzVault, ChemSpider (www.chemspider.com), and Human Metabolome Database (HMDB, www.hmdb.ca) in combination with accurate molecular mass and MS/MS information. Statistical analyses included both multivariate approaches and univariate methods for differential expression analysis, to identify features with significant differences between groups. Multivariate statistical analyses, including Principal Component Analysis (PCA) and Orthogonal Projections to Latent Structures-Discriminant Analysis (OPLS-DA), were performed using SIMCA-P software (version 14.1, Umetrics, Sweden). Differential expression analysis was performed using the *MetaboAnalystR* package in R (version 4.4.2) with significance thresholds set at *P* < 0.05 and *|log*_*2*_ *FC|* ≥ 1.2. Visualization of the results was achieved by generating volcano plots using the *ggplot2* package. Heatmaps were generated using the *pheatmap* package. The *pROC* package was used to plot ROC curves and calculate AUC values. During data analysis of metabolite peak areas from targeted metabolomics, normally distributed data were analyzed using Student’s *t*-test or ANOVA, while non-normally distributed data were analyzed with the Mann-Whitney U test (SPSS Statistics v25). Visualizations were created in GraphPad Prism v8.0.2 and refined in Adobe Illustrator v26.0.

## Results

### Results of the non-targeted metabolomic study

**Fig. 1:**
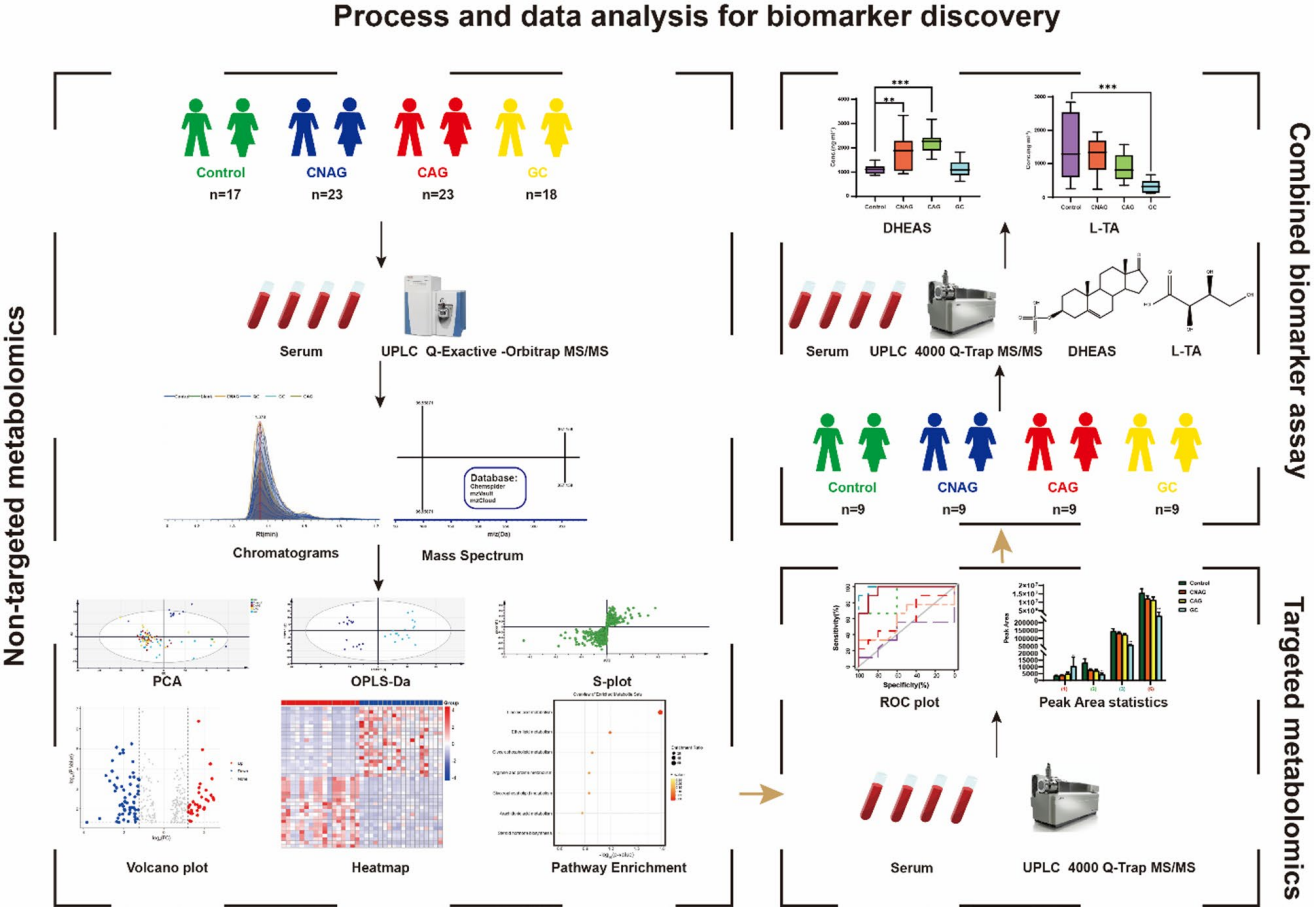
Workflow of biomarker discovery and validation.

**Fig. 2:**
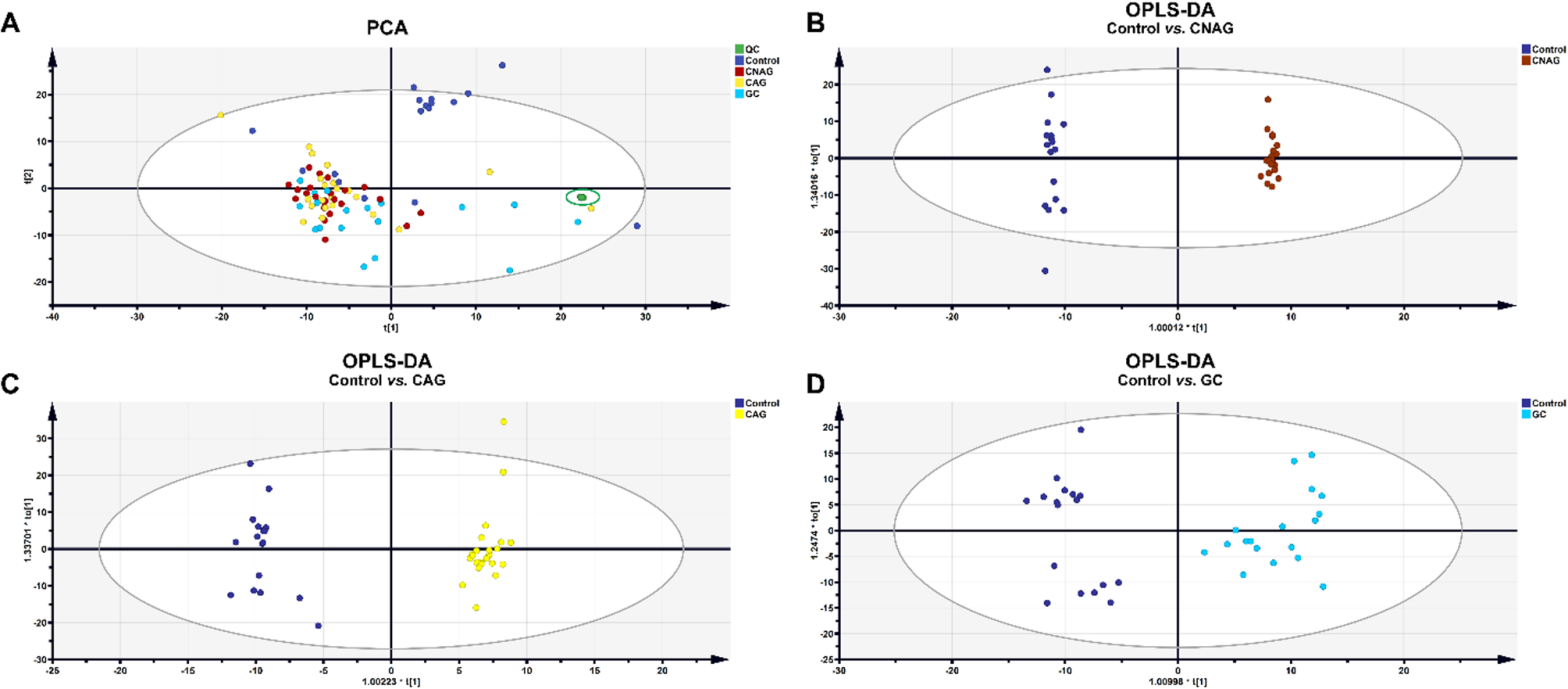
Results of the multivariate analysis of UPLC-HRMS data (non-targeted metabolomics). (**A**) PCA score plot for QC, Control, CNAG, CAG, and GC groups. (**B**) OPLS-DA score plot for the Control vs. CNAG groups. (**C**) OPLS-DA score plot for the Control vs. CAG groups. (**D**) OPLS-DA score plot for the Control vs. GC groups.

The workflow diagram for biomarker discovery and validation is shown in Fig. 1. Compound Discoverer software and multiple database searches were used for structure prediction for both positive and negative ion modes. Totals of 1,570 and 1,433 compounds (exact molecular weights) were detected in these modes, respectively. However, a total of 763 endogenous metabolites were identified after data collation. And subsequently, we performed multivariate statistical analysis of these metabolites. The typical identification workflow for compounds is illustrated in Fig. S1. The total ion chromatogram curves for the various groups in both positive and negative ion modes included overlapping peaks with consistent retention times and peak intensities, indicating good signal stability throughout the analytic process (Fig. S2). PCA score plots were generated for raw data analysis (Fig. 2A), and the QC samples were closely clustered, indicating stable instrument conditions during the sample data acquisition phase, which should ensure the accuracy and reliability of the analytical data. There was clear separation between the control group and the disease groups, which suggested that there were variations in the concentrations of certain endogenous metabolites during disease progression. Moreover, as the severity of the disease increased, the separation between the control group and the disease groups became more pronounced.

A supervised method of multivariate statistical analysis, OPLS-DA, was employed to maximize intergroup separation. In this, the X-axis represents the predictive principal component, indicating intergroup differences along the horizontal direction, while the Y-axis represents the orthogonal principal component, and therefore displays within-group differences in the vertical dimension. Three separate OPLS-DA models were established by comparing the control group with the CNAG, CAG, and GC groups (Fig. 2B-D). To validate the robustness of the OPLS-DA model and to assess potential overfitting, a permutation test (200 random permutations) was performed. In this case: R² (goodness of fit) represents the proportion of variance explained by the model. Q² (predictability) represents the predictive power of the model as estimated by cross-validation. The results showed that both R² and Q² values were close to 1.0, confirming the strong explanatory power and predictive reliability of the model, and that the intercept of the Q² regression line was negative (< 0), with a low risk of overfitting. The OPLS-DA score plot for the GC vs. the CNAG and CAG groups are shown in the Fig S3A. Based on the OPLS-DA model, the variable importance in the projection (VIP), which is an indicator of the metabolite’s importance (significance of the differences) in the model, was determined for each group.

**Fig. 3:**
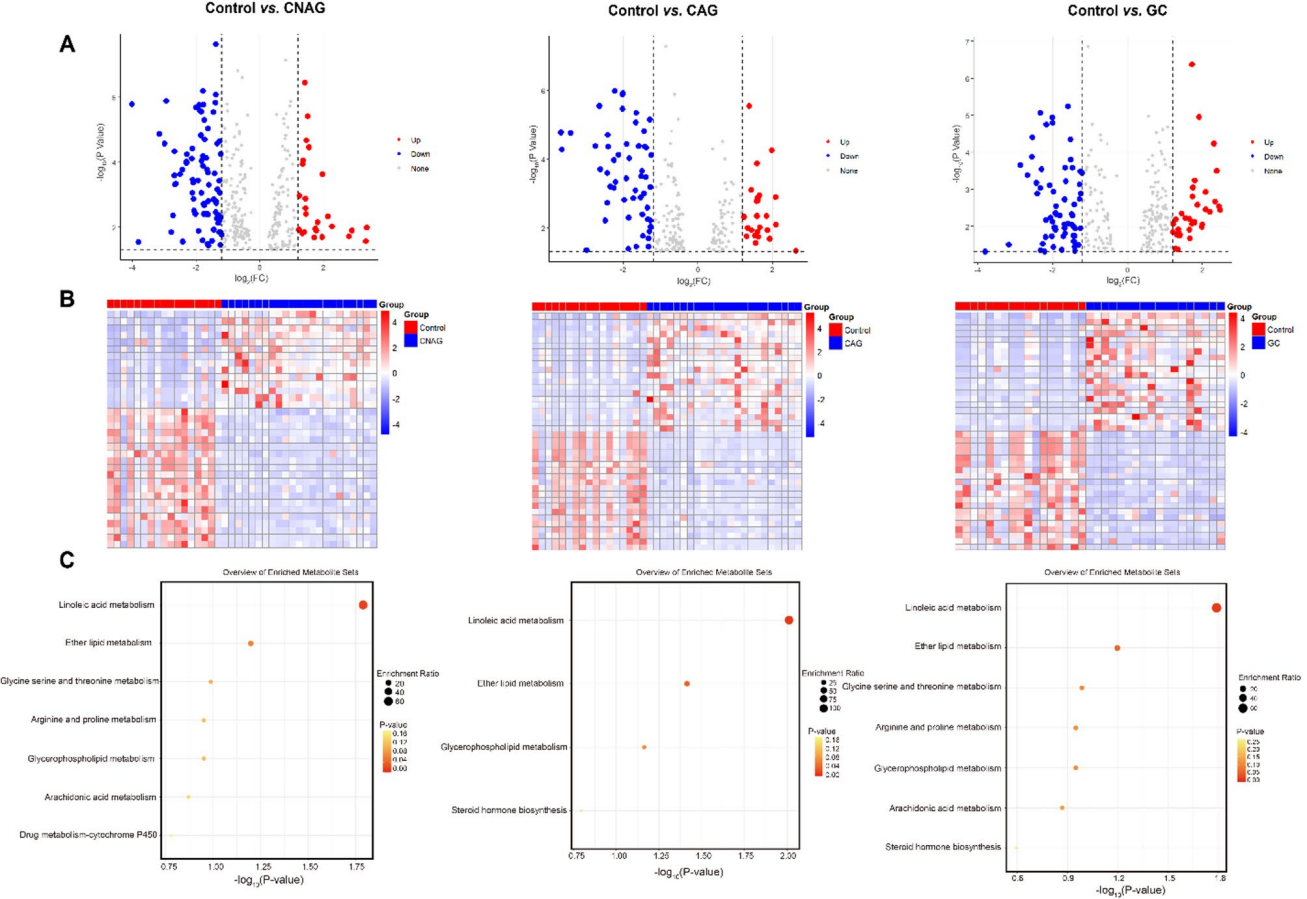
Metabolites present at differing concentrations (DMs) on non-targeted metabolomics. (**A**) Volcano plot of DMs for the Control vs. CNAG, CAG, and GC groups, to identify DMs. (**B**) Heatmap plot of the DMs for the Control vs. CNAG, CAG, and GC groups. (**C**) Results of the pathway enrichment analysis of the DMs.

Subsequently, *t*-tests and the calculation of FC were performed using the relative concentrations (peak areas) of the metabolites in the Control, CNAG, CAG, and GC groups. Potential differential metabolites were visualized by volcano plots (Fig. 3A). The potential differential metabolites that met the criteria were sorted according to *P* < 0.05, and the up-regulated and down-regulated Top20 differential metabolites of each group were taken separately, and the intersection with the differential metabolites with VIP > 1 was taken for the heatmap, which was to present the differences in the concentrations of the significant differential metabolites among the groups in a more intuitive way (Fig. 3B). Fig S3 B-C display the volcano plot and heatmap plot of the DMs for the GC vs. the CNAG and CAG groups.

Finally, the identified metabolites were subjected to pathway enrichment analysis using the KEGG database^26–28^ (Fig. 3C). The disease groups showed abnormalities in metabolic pathways, including linoleic acid metabolism, ether lipid metabolism, glycine, serine and threonine metabolism, arginine and proline metabolism, glycerophospholipid metabolism, arachidonic acid metabolism, steroid hormone biosynthesis, and drug metabolism - cytochrome P450. These abnormalities would primarily affect amino acid metabolism, lipid metabolism, and microbial metabolism. Notably, linoleic acid metabolism, ether lipid metabolism, and glycerophospholipid metabolism were found to be abnormal in all three disease groups; thus, further investigation is warranted.

### Differential metabolite profiling and validation in targeted metabolomics analysis

To further characterize the structures and quantify the differences in the concentrations of metabolites of interest, the metabolites were carefully selected from the disease groups on the basis of the results of non-targeted metabolomics. Subsequently, a LRMS technique employing MRM ion pairs was established, which was capable of quantifying 32 metabolites in positive ion mode and 24 metabolites in negative ion mode. Using the established UPLC-MS-MRM method, serum samples from 57 participants that had previously been analyzed using non-targeted metabolomics were re-analyzed. The peak areas of the target ion pairs for each sample were quantified in a single batch. The results indicated that a total of 56 metabolites could be detected in positive or negative ion mode.

**Fig. 4:**
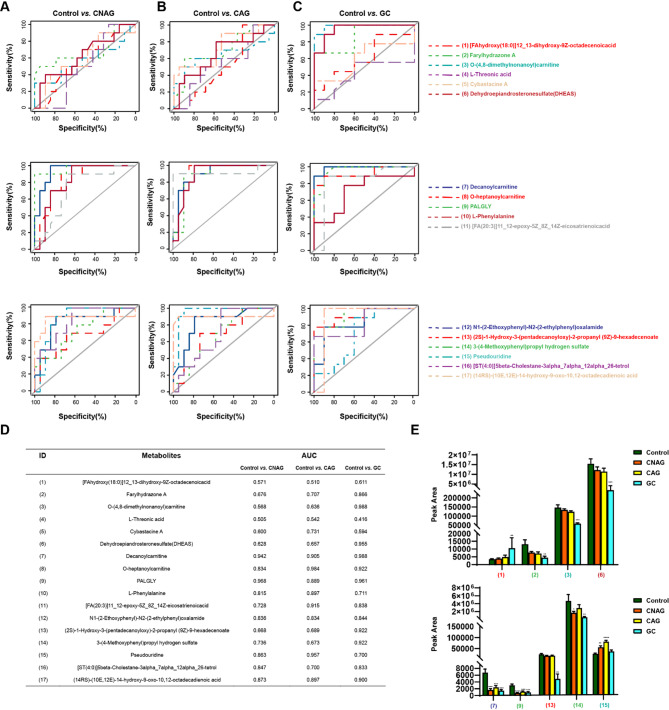
Metabolites present at differing concentrations (DMs) in targeted metabolomics. (**A**) ROC curves for typical DMs for the Control vs. CNAG groups; (**B**) the Control vs. CAG groups; and (**C**) the Control vs. GC groups; (**D**) Names and AUCs of the selected DMs in the different groups; (**E**) Statistical plots of the mean peak areas for the various groups with respect to the DMs identified using the UPLC-MS-MRM assay. Control (*n* = 10), CNAG (*n* = 19), CAG (*n* = 19), and GC (*n* = 9). Control vs. Disease group *P < 0.05, **P < 0.01 and ***P < 0.001, Student’s *t*-test.

In order to further validate the metabolites of interest, ROC curves were generated for the chromatographic peak areas of all the newly identified metabolites, and the associated AUC values were calculated. The ROC curve analysis showed that 17 metabolites had AUC values > 0.5 in the Control vs. CNAG, Control vs. CAG, and Control vs. GC groups (Fig. 4A–D). The AUC values for metabolites in the GC versus CNAG and CAG groups are summarized in Table S3. Of these, six metabolites showed changes in concentration with disease progression. There were significant inter-group differences in the peak areas for O-(4,8-dimethylnonanoyl) carnitine (3) and DHEAS (6), but the other metabolites did not show consistent trends or significant differences between the disease groups (Fig. 4E).

**Fig. 5:**
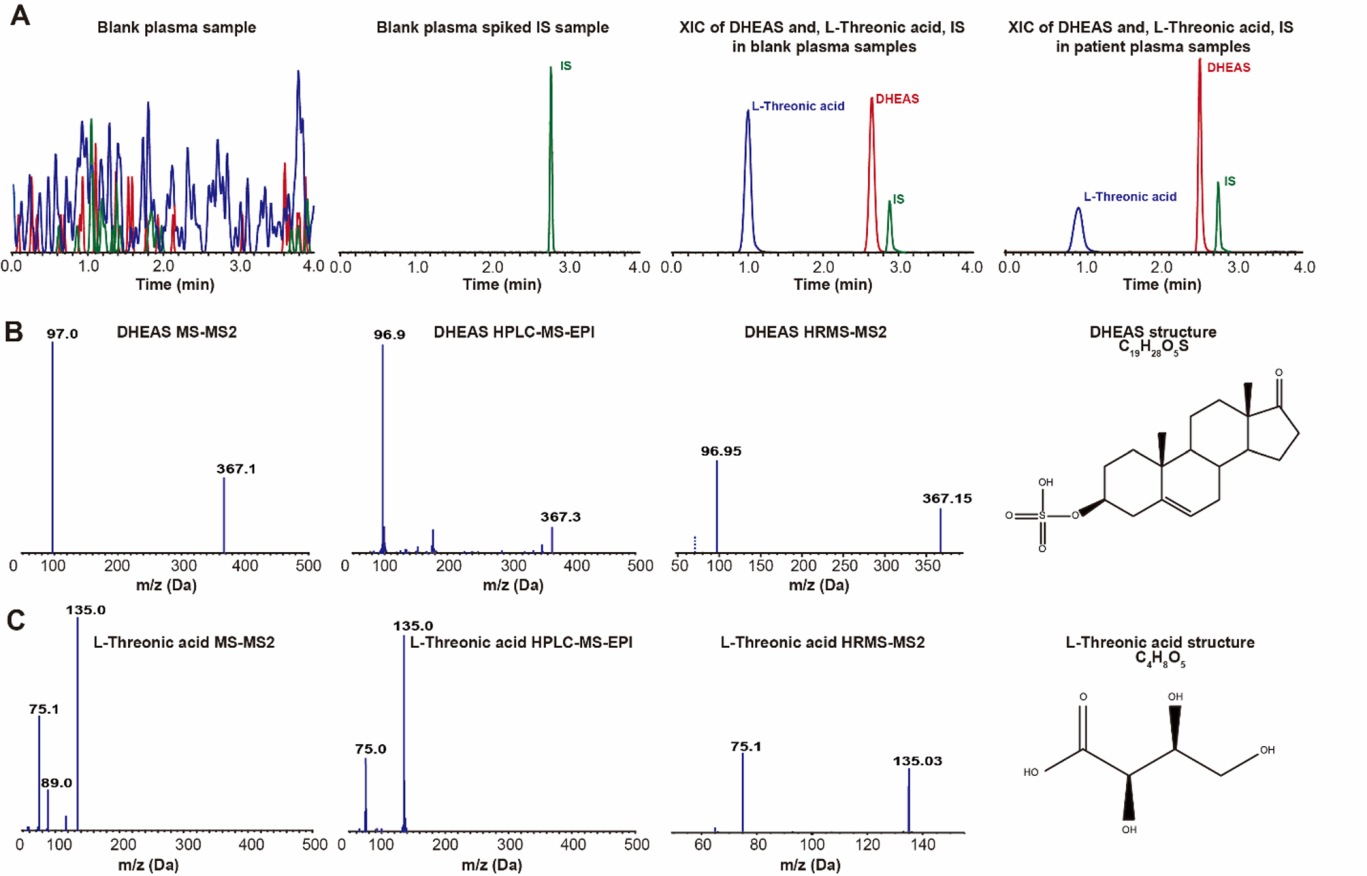
Chromatographic, mass spectrometry, and structural information for the potential biomarkers DHEAS and L-TA. (**A**) Extracted ion chromatograms for DHEAS and L-TA in simulated blank serum and participant-derived serum; (**B**) Structure of DHEAS and its secondary mass spectra (MS2) derived from the standard, LRMS (UPLC-MRM-EPI), and HRMS (UPLC-Full Scan-ddMS2) analyses of the serum samples; (**C**) Structure of L-TA and its secondary mass spectra (MS2) derived from standard, LRMS (UPLC-MRM-EPI), and HRMS (UPLC-Full Scan-ddMS2) analyses of the serum samples.

Based on a comprehensive evaluation of factors including differential significance across groups, analytical stability, commercial availability of standards, physiological concentrations in vivo, and feasibility for clinical detection, we selected the metabolites L-TA and DHEAS for further validation of their chemical structures. The DHEAS standard predominantly generated a quasi-molecular ion peak [M-H]^−^ at m/z 367.1 in negative ion mode, with electrospray ionization (ESI). Chromatographic analysis demonstrated that the retention time (t_R_) of the standard was consistent with the t_R_ of the ion pair extracted from serum samples, with t_R_=26.5 min (Fig. 5A). The secondary mass spectrometry (MS2) ion fragments produced by the DHEAS standard matched those generated by both LRMS with MRM-enhanced product ion (MRM-EPI) mode and HRMS with Full scan-ddMS2 mode for the serum samples from the participants (Fig. 5B). Similarly, the chromatographic retention time and MS characteristics of the L-TA standard were consistent with those obtained using the serum samples (Fig. 5C). These findings provided conclusive evidence for the structures of both compounds.

**Fig. 6:**
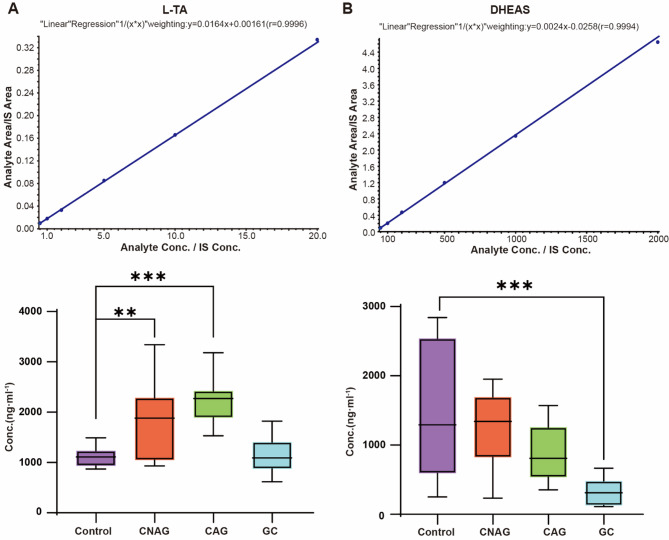
Standard curve for the validation of a method for the simultaneous assay of DHEAS and LTA. (**A**) Standard curve for DHEAS and L-TA in simulated serum. (**B**) Concentrations of DHEAS and L-TA in new serum samples from participants in the Control, CNAG, CAG, and GC groups. Control (*n* = 9), CNAG (*n* = 9), CAG (*n* = 9) and GC (*n* = 9). *P < 0.05, **P < 0.01, and ***P < 0.001.

We next aimed to establish a method for the simultaneous determination of the DHEAS and L-TA concentrations in human serum samples, involving the assessment of the specificity, standard curve, limits of quantification (LOQs), precision, recovery, stability, and matrix effects. The generated method showed good specificity and there was no significant interference associated with the simultaneous determination of DHEAS and L-TA in serum samples (Fig. 5A). DHEAS exhibited excellent Linearity within a concentration range of 50 to 2,000 ng/ml (*r* > 0.999), with a standard curve equation of y = 0.0024x − 0.0258. Similarly, the method developed demonstrated robust Linearity for L-TA within a concentration range of 500 to 20,000 ng/ml (*r* > 0.999), with a standard curve equation of y = 0.0164x + 0.00161. The precision and recovery rates met the required thresholds, no significant matrix effects were observed (Table 1; Fig. 6), and the LOQs for the two components were 50 ng/ml and 100 ng/ml, respectively. In addition, the samples were stable at room temperature for 2 h, − 80 °C for 1 month, and after undergoing three freeze–thaw cycles.

We also collected additional serum samples from 36 participants (*n* = 9 samples per group) and quantitatively assayed the target metabolites using the developed method. The results demonstrated higher L-TA concentrations in the CNAG and CAG groups than in the Control group. Conversely, the DHEAS concentration gradually decreased with disease progression, and there were significant differences between the Control and GC groups and between the CNAP and GC groups (Fig. 6). The distinct trends in the concentrations of these metabolites were consistent with the results of the metabolomics studies.

**Table 1:** Validation of a method for the simultaneous determination of DHEAS and L-TA in human serum, including the precision, accuracy, matrix effect, and extraction recovery (*n* = 6).

Analyte	Concentration (ng/ml)	RSD (%)		Accuracy (%)	Mean matrix effect (%)	Mean extraction recovery (%)
	Add	Intra-day	Inter-day			
DHEAS	80	4.52	5.90	104.93	100.47	109.57
	400	3.61	0.37	96.07	97.91	100.30
	1600	2.91	4.63	109.41	102.69	90.92
L-TA	600	6.29	2.77	98.62	111.00	109.00
	3000	5.53	5.50	99.54	96.80	110.00
	15,000	6.49	6.80	98.30	91.50	101.00

## Discussion

GC is characterized by high incidence and mortality rates, which underscores the importance of an early diagnosis for a good treatment outcome. CNAG and CAG are prevalent gastric diseases and precursors to gastric cancer, which is evidenced by the fact that > 80% of patients with GC exhibit gastric mucosal atrophy^13^. Therefore, it is important to identify reliable biomarkers of GC, and especially ones that reflect the progression from CNAG to CAG and GC. In the present study, serum samples were collected from healthy individuals and patients with CNAG, CAG, or GC, and through both non-targeted and targeted metabolomic approaches, DHEAS and L-TA were identified to be present at significantly different concentrations in the various groups. Subsequently, a method of simultaneously determining the concentrations of these biomarkers in human serum was established and used to measure the concentrations of these substance in samples collected from some of the same participants. In this way, we were able to confirm the significant differences in concentrations among the groups. Taken together, these findings support the potential utility of the identified metabolites for use as biomarkers for the early diagnosis of, and risk assessment of patients with, CNAG, CAG, or GC.

Metabolomic studies that utilize high-resolution mass spectrometry are powerful tools for the identification of biomarkers. However, most studies have used non-targeted metabolomics and focused on the identification of metabolites present at differing concentrations and bioinformatic analysis of related metabolic pathways, but they often lack the necessary validation^29–32^. Owing to uncertainties in compound (metabolite) structure identification relating to the characteristics of high-resolution mass spectrometry and the subsequent data processing, the results of non-targeted metabolomics alone are not directly applicable to clinical diagnosis and treatment. In the present study, we addressed this limitation by selecting metabolites that were present at significantly different concentrations and performing high-quality secondary mass spectrometry. MRM ion pairs were established and reanalyzed using a triple quadrupole low-resolution mass spectrometer, which enabled the validation of the structures and the differences in their concentrations using targeted metabolomics. This method was based on obtaining ion pair information regarding metabolites using high-resolution mass spectrometry, filtering out secondary fragment mass spectrometry peaks with low responses, and then using targeted multi-reaction detection to measure metabolite abundance. This method does not require standard samples or internal standards, the use of which limits the number of metabolites analyzed, and is associated with a high level of coverage and good repeatability^33–35^. Finally, the structures of the selected potential biomarkers, DHEAS and L-TA, were confirmed through the purchase of standard compounds.

In the present study, we conducted a systematic investigation that encompassed the confirmation of the identity of the candidate biomarkers and their potential clinical applications, with respect to CNAG, CAG, and GC. Other identified metabolites, such as O-(4,8-dimethylnonanoyl) carnitine (3), decanoylcarnitine (7), and (2 S)−1-hydroxy-3-(pentadecanoyloxy)−2-propanyl (9Z)−9-hexadecenoate (13), exhibited greater reliability and more significant inter-group differences, according to their AUC curves (Fig. 5D), and therefore they may be more suitable as biomarkers for diagnostic purposes. However, because standard reference compounds for these metabolites are not commercially available, their utility requires further investigation and validation.

The quantitative analysis of small molecule metabolites is primarily performed using chemical methods, such as chromatography, mass spectrometry, and nuclear magnetic resonance (NMR). Of these, liquid chromatography is the most commonly utilized technique, owing to its cost-effectiveness and widespread availability. There are challenges associated with the use of liquid chromatography in combination with ultraviolet or fluorescence detectors for the analysis of complex biological samples, including difficulties with separation and low sensitivity. However, owing to the high sensitivity and specificity of mass spectrometers, they are being increasingly utilized for clinical diagnostics. Using the newly developed and validated UPLC-MS/MS method for simultaneous assay of DHEAS and L-TA in human serum, clinical samples require only simple protein precipitation, and the DHEAS and L-TA concentrations can be accurately quantified within 4 min. The consistent differences identified in the serum concentrations of the two biomarkers (DHEAS and L-TA) between the disease groups and the control group suggests that they could be used reliably to make diagnoses. Given the ease with which serum samples can be obtained and the high compliance associated, this method represents a promising auxiliary diagnostic method that should facilitate the early detection of GC.

**Fig. 7:**
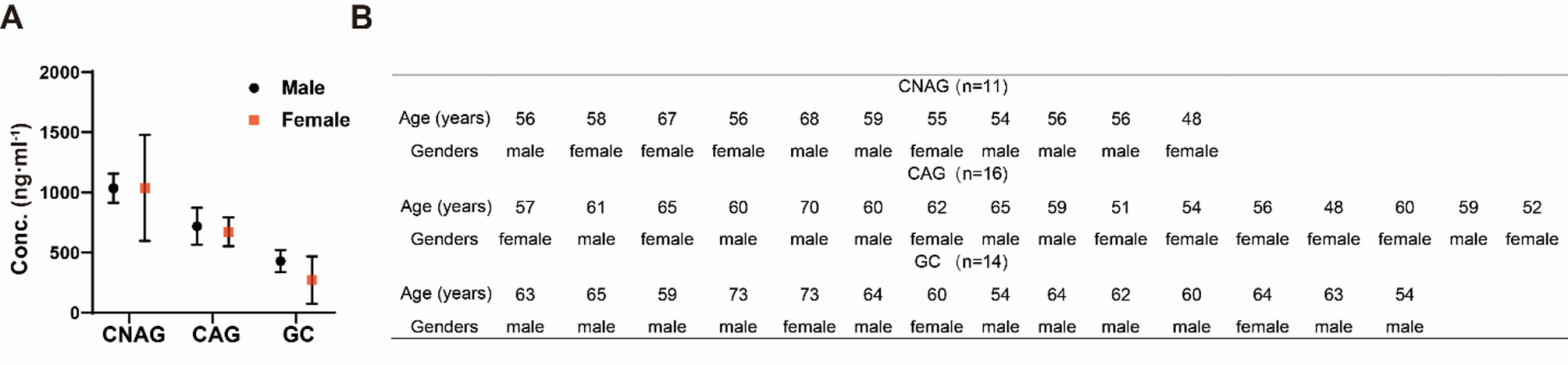
Concentrations of the potential biomarker DHEAS in participants of specific sexes and ages. (**A**) Bar chart of the concentration of DHEAS in participants in the CNAG, CAG, and GC groups. (**B**) Information regarding the age and sex of the participants in each disease group. CNAG (*n* = 11), CAG (*n* = 16), and GC (*n* = 14).

The identification of specific biomarkers assists with our understanding of disease-related mechanisms and may become potential targets for drug therapy. Dehydroepiandrosterone (DHEA), which is primarily secreted by the adrenal glands, but is also synthesized in the central nervous system, is present at high concentrations in human plasma. Although specific nuclear receptors have not been identified, the pharmacologic effects of DHEA, along with its sulphate ester (DHEAS), have been extensively studied. Previous studies have shown a close association between low DHEA/S concentrations and the risks of cardiovascular disease and type 2 diabetes^36^. Conversely, high DHEA/S concentrations or supplementation are associated with immune-modulating, anti-obesity, anti-cancer, anti-osteoporosis, anti-aging, and other effects^36, 37^. In the present study, DHEA/S demonstrated significant disease progression-associated changes in concentration across the various disease groups. The concentration gradually decreased with increases in the severity of the disease, such that it was less than a third of that of the controls, implying that this may be clinically useful parameter. According to previous studies^38–41^the DHEAS concentration is associated with sex and age. To assess the potential influence of these variables on current findings, we analyzed samples from participants aged 45–75 years with demographically balanced sex distribution across age groups for DHEAS concentration-related statistical analyses (Fig. 7). Disease progression-associated variations in DHEAS concentrations were preserved in this analysis and were not confounded by age or sex factors. While males exhibited higher DHEAS concentrations compared to females, this observation aligns with established biological patterns. However, the insufficient sample size precluded stratified comparative analysis of DHEAS concentrations between disease cohorts and control groups.

L-TA, a natural sugar acid that is found in the human body, has been associated with bone health and has effects on the central nervous system^42–47^. Recently, L-TA was identified through non-targeted metabolomics as a potential biomarker of acute myocardial ischemia (AMI)^48^. In the present study, L-TA showed a gradual increase from the control to the CNAG and CAG groups; however, the concentrations in the GC group did not significantly differ from those of controls. The molecular links of DHEAS and L-TA with CNAG, CAG, and GC require further exploration. This information could provide valuable insight into these diseases and aid in the discovery of drug treatment targets.

There are some Limitations of this study. We reacquired and analyzed clinical data from 36 cases, and significant differences in assay and biomarker profiles were validated through this investigation. While these findings have methodological implications for understanding disease mechanisms and demonstrate potential clinical applicability in diagnosis and risk assessment, the relatively limited sample size calls for future validation through extensive multicenter studies. Our targeted metabolomics approach using triple quadrupole mass spectrometry-based assays improved the specificity of the DHEAS and L-TA assays, but has inherent limitations in terms of cross-platform reproducibility. In addition to the significance of differences, the selection of biomarkers needs to take into account the stability of candidates, the availability of standards, the in vivo concentration of substances, and the maneuverability of clinical assays, etc. In this study, we finally selected DHEAS and L-TA as potential biomarkers after comprehensive consideration. Although we have fully demonstrated that L-TA is significantly elevated in precancerous lesions, yet its level is not significantly different from the normal group in cancer patients, and these features can be used to differentiate between different gastric diseases, but the exact mechanism is still unclear. To address these limitations, in a follow-up study we need to systematically re-collect samples and perform clinical validation assays. This aims to establish standardized reference ranges for serum DHEAS and L-TA concentrations for healthy controls and pathological groups (CNAG, CAG, GC) and to perform rigorous multicenter validation of clinically actionable biomarker thresholds. In addition, we will continue to explore the possibility of other differential metabolites as biomarkers and the potential value of these substances in explaining the pathogenesis of CNAG, CAG, and GC.

## Conclusions

In the present study, non-targeted and targeted metabolomics approaches were used to characterize the metabolites profiles of serum samples obtained from clinically normal individuals and patients who had been diagnosed with CNAG, CAG, or GC. Two potential biomarkers, DHEAS and L-TA, were successfully identified and validated as being strongly associated with disease progression. A method for the simultaneous determination of these potential biomarkers was developed, which may provide a valuable tool for the early diagnosis and clinical risk assessment of CNAG, CAG, and GC. The presence of a low DHEAS concentration in the absence of a high L-TA concentration may represent an early warning sign for GC.

## Supplementary Information

Below is the link to the electronic supplementary material.

Supplementary Material 1

## Data Availability

The mass spectrometry metabolomics data generated in this study has been deposited in the MetaboLights database under the unique identifier MTBLS12186. The data can be accessed at the following link: https://www.ebi.ac.uk/metabolights/reviewer7c2c3032-91f3-4b14-9270-8b0a684c42c5.

## Abbreviations

AMI: Acute myocardial ischemia
AUC: Area under curve
CAG: Chronic atrophic gastritis
CNAG: Chronic non-atrophic gastritis
DHEA: Dehydroepiandrosterone
DHEAS: Dehydroepiandrosterone sulfate
DMs: Differential metabolites
EPI: Enhanced product ion
ESI: Electrospray ionization
FDR: False discovery rate
GC: Gastric cancer
HRMS: High-resolution mass spectrometer
IS: Internal standard
LRMS: Low-resolution mass spectrometer
MRM: Multiple reaction monitoring
MS1: Mass spectrum
MS2: The secondary mass spectrum
NMR: Nuclear magnetic resonance
OPLS-DA: Orthogonal partial least squares discriminant analysis
PCA: Principal component analysis
QC: Quality control
ROC: Receiver operating characteristic
UPLC–MS/MS: Ultra-high performance liquid chromatography–tandem mass spectrometry
VIP: Variables of importance in the project

## References

[1] Smyth, E. C., Nilsson, M., Grabsch, H. I., van Grieken, N. C. & Lordick, F. *Gastric Cancer Lancet* **396**, 635–648 (2020). 32861308 10.1016/S0140-6736(20)31288-5

[2] Ferlay, J. et al. Estimating the global cancer incidence and mortality in 2018: Globocan sources and methods. *Int. J. Cancer*. **144**, 1941–1953 (2019). 30350310 10.1002/ijc.31937

[3] Ajani, J. A. et al. Gastric cancer, version 2.2022, Nccn clinical practice guidelines in oncology. *J. Natl. Compr. Cancer Netw.* **20**, 167–192 (2022). 10.6004/jnccn.2022.0008 35130500

[4] He, J. et al. [China guideline for the screening, early detection and early treatment of esophageal cancer (2022, Beijing)]. *Zhonghua Zhong Liu Za Zhi*. **44**, 491–522 (2022). 35754225 10.3760/cma.j.cn112152-20220517-00348

[5] Li, K., Zhang, A., Li, X., Zhang, H. & Zhao, L. Advances in clinical immunotherapy for gastric cancer. *Biochim. Biophys. Acta-Rev Cancer*. **1876**, 188615 (2021). 34403771 10.1016/j.bbcan.2021.188615

[6] Thrift, A. P. & El-Serag, H. B. Burden of gastric cancer. *Clin. Gastroenterol. Hepatol.* **18**, 534–542 (2020). 31362118 10.1016/j.cgh.2019.07.045 PMC8859863

[7] Bacha, D. et al. Chronic gastritis classifications. *Tunis Med.* **96**, 405–410 (2018). 30430483

[8] Marques-Silva, L., Areia, M., Elvas, L. & Dinis-Ribeiro, M. Prevalence of gastric precancerous conditions: A systematic review and Meta-Analysis. *Eur. J. Gastroenterol. Hepatol.* **26**, 378–387 (2014). 24569821 10.1097/MEG.0000000000000065

[9] Chinese Society of Gastroenterology. G. O. Guidelines for diagnosis and treatment of chronic gastritis in China (2022, Shanghai). *J. Dig. Dis.* **24**, 150–180 (2023). 37245073 10.1111/1751-2980.13193

[10] Fang, J. Y. et al. Chinese consensus on chronic gastritis (2017, Shanghai). *J. Dig. Dis.* **19**, 182–203 (2018). 29573173 10.1111/1751-2980.12593

[11] Li, Y., Xia, R., Zhang, B. & Li, C. Chronic atrophic gastritis: A review. *J. Environ. Pathol. Toxicol. Oncol.* **37**, 241–259 (2018). 30317974 10.1615/JEnvironPatholToxicolOncol.2018026839

[12] Lim, N. R. & Chung, W. C. Helicobacter Pylori-Associated chronic atrophic gastritis and progression of gastric carcinogenesis. *Korean J. Gastroenterol.* **82**, 171–179 (2023). 37876256 10.4166/kjg.2023.097 PMC12285364

[13] Sipponen, P., Kekki, M., Haapakoski, J., Ihamaki, T. & Siurala, M. Gastric cancer risk in chronic atrophic gastritis: statistical calculations of Cross-Sectional data. *Int. J. Cancer*. **35**, 173–177 (1985). 3871738 10.1002/ijc.2910350206

[14] Correa, P. Human gastric carcinogenesis: A multistep and multifactorial Process–First American cancer society award lecture on cancer epidemiology and prevention. *Cancer Res.* **52**, 6735–6740 (1992). 1458460

[15] Yin, Y., Liang, H., Wei, N. & Zheng, Z. Prevalence of chronic atrophic gastritis worldwide from 2010 to 2020: an updated systematic review and Meta-Analysis. *Ann. Palliat. Med.* **11**, 3697–3703 (2022). 36635994 10.21037/apm-21-1464

[16] Correa, P. & Piazuelo, M. B. The gastric precancerous cascade. *J. Dig. Dis.* **13**, 2–9 (2012). 22188910 10.1111/j.1751-2980.2011.00550.x PMC3404600

[17] Johnson, C. H., Ivanisevic, J. & Siuzdak, G. Metabolomics: beyond biomarkers and towards mechanisms. *Nat. Rev. Mol. Cell. Biol.* **17**, 451–459 (2016). 26979502 10.1038/nrm.2016.25 PMC5729912

[18] Wishart, D. S. Metabolomics for investigating physiological and pathophysiological processes. *Physiol. Rev.* **99**, 1819–1875 (2019). 31434538 10.1152/physrev.00035.2018

[19] Schrimpe-Rutledge, A. C., Codreanu, S. G., Sherrod, S. D. & McLean, J. A. Untargeted metabolomics Strategies-Challenges and emerging directions. *J. Am. Soc. Mass. Spectrom.* **27**, 1897–1905 (2016). 27624161 10.1007/s13361-016-1469-y PMC5110944

[20] de Mello, R. A. et al. Current and potential biomarkers in gastric cancer: A critical review of the literature. *Future Oncol.* **17**, 3383–3396 (2021). 34291647 10.2217/fon-2021-0084

[21] Rocken, C. Predictive biomarkers in gastric cancer. *J. Cancer Res. Clin. Oncol.* **149**, 467–481 (2023). 36260159 10.1007/s00432-022-04408-0 PMC9889517

[22] Kadam, W., Wei, B. & Li, F. Metabolomics of gastric cancer. *Adv. Exp. Med. Biol.* **1280**, 291–301 (2021). 33791990 10.1007/978-3-030-51652-9_20

[23] Fan, Y. & Pedersen, O. Gut microbiota in human metabolic health and disease. *Nat. Rev. Microbiol.* **19**, 55–71 (2021). 32887946 10.1038/s41579-020-0433-9

[24] Liu, J. et al. Integrative metabolomic characterisation identifies altered portal vein serum metabolome contributing to human hepatocellular carcinoma. *Gut* **71**, 1203–1213 (2022). 34344785 10.1136/gutjnl-2021-325189 PMC9120406

[25] Alseekh, S. et al. Mass Spectrometry-Based metabolomics: A guide for annotation, quantification and best reporting practices. *Nat. Methods*. **18**, 747–756 (2021). 34239102 10.1038/s41592-021-01197-1 PMC8592384

[26] Kanehisa, M., Furumichi, M., Sato, Y., Matsuura, Y. & Ishiguro-Watanabe, M. Kegg: biological systems database as a model of the real world. *Nucleic Acids Res.* **53**, D672–D677 (2025). 39417505 10.1093/nar/gkae909 PMC11701520

[27] Kanehisa, M. Toward Understanding the origin and evolution of cellular organisms. *Protein Sci.* **28**, 1947–1951 (2019). 31441146 10.1002/pro.3715 PMC6798127

[28] Kanehisa, M., Goto, S. & Kegg Kyoto encyclopedia of genes and genomes. *Nucleic Acids Res.* **28**, 27–30 (2000). 10592173 10.1093/nar/28.1.27 PMC102409

[29] Pan, C. et al. Metabolomics study identified bile acids as potential biomarkers for gastric cancer: A case control study. *Front. Endocrinol.* **13**, 1039786 (2022). 10.3389/fendo.2022.1039786 PMC9715751 36465663

[30] Pavlova, N. N., Zhu, J. & Thompson, C. B. The hallmarks of cancer metabolism: still emerging. *Cell. Metab.* **34**, 355–377 (2022). 35123658 10.1016/j.cmet.2022.01.007 PMC8891094

[31] Sun, K. et al. Plasma metabolic signatures for intracranial aneurysm and its rupture identified by pseudotargeted metabolomics. *Clin. Chim. Acta*. **538**, 36–45 (2023). 36347333 10.1016/j.cca.2022.11.002

[32] Wang, M. et al. Discovery of plasma biomarkers for colorectal cancer diagnosis via untargeted and targeted quantitative metabolomics. *Clin. Transl Med.* **12**, e805 (2022). 35389561 10.1002/ctm2.805 PMC8989079

[33] Chen, S. et al. Pseudotargeted metabolomics method and its application in serum biomarker discovery for hepatocellular carcinoma based on ultra High-Performance liquid chromatography/triple quadrupole mass spectrometry. *Anal. Chem.* **85**, 8326–8333 (2013). 23889541 10.1021/ac4016787

[34] Wei, Y. et al. Early breast cancer detection using untargeted and targeted metabolomics. *J. Proteome Res.* **20**, 3124–3133 (2021). 34033488 10.1021/acs.jproteome.1c00019

[35] Roberts, L. D., Souza, A. L., Gerszten, R. E. & Clish, C. B. Targeted metabolomics. *Curr Protoc. Mol. Biol Chapter*. **30**, 30–32 (2012). 10.1002/0471142727.mb3002s98 PMC3334318 22470063

[36] Aoki, K. & Terauchi, Y. Effect of dehydroepiandrosterone (dhea) on diabetes mellitus and obesity. *Vitam. Horm.* **108**, 355–365 (2018). 30029734 10.1016/bs.vh.2018.01.008

[37] Nenezic, N. et al. Dehydroepiandrosterone (dhea): Pharmacological effects and potential therapeutic application. *Mini-Rev Med. Chem.* **23**, 941–952 (2023). 36121077 10.2174/1389557522666220919125817

[38] Elprince, M., Kishk, E. A., Metawie, O. M. & Albiely, M. M. Ovarian stimulation after dehydroepiandrosterone supplementation in poor ovarian reserve: A randomized clinical trial. *Arch. Gynecol. Obstet.* **302**, 529–534 (2020). 32451660 10.1007/s00404-020-05603-5

[39] Sahu, P., Gidwani, B. & Dhongade, H. J. Pharmacological activities of dehydroepiandrosterone: A review. *Steroids* **153**, 108507 (2020). 31586606 10.1016/j.steroids.2019.108507

[40] Nagaya, T., Kondo, Y. & Okinaka, T. Serum Dehydroepiandrosterone-Sulfate reflects age better than health status, and May increase with cigarette smoking and alcohol drinking in Middle-Aged men. *Aging Clin. Exp. Res.* **24**, 134–138 (2012). 22842832 10.1007/BF03325159

[41] Stomati, M. et al. Six-Month oral dehydroepiandrosterone supplementation in early and late postmenopause. *Gynecol. Endocrinol.* **14**, 342–363 (2000). 11109974 10.3109/09513590009167703

[42] He, J. H., Tong, N. W., Li, H. Q. & Wu, J. [Effects of L-Threonate on bone resorption by osteoclasts in vitro]. *Sichuan Da Xue Xue Bao Yi Xue Ban*. **36**, 225–228 (2005). 15807273

[43] Wang, H., Hu, P. & Jiang, J. Calcium bioavailability of calcium L-threonate in healthy Chinese subjects measured with stable isotopes (⁴⁴ca and ⁴²Ca). *Eur. J. Clin. Pharmacol.* **69**, 1121–1126 (2013). 23229796 10.1007/s00228-012-1420-5

[44] Kwack, M. H., Ahn, J. S., Kim, M. K., Kim, J. C. & Sung, Y. K. Preventable effect of L-Threonate, an ascorbate metabolite, on Androgen-Driven balding via repression of Dihydrotestosterone-Induced Dickkopf-1 expression in human hair dermal papilla cells. *Bmb Rep.* **43**, 688–692 (2010). 21034532 10.5483/BMBRep.2010.43.10.688

[45] Li, W. et al. Elevation of brain magnesium prevents synaptic loss and reverses cognitive deficits in alzheimer’s disease mouse model. *Mol. Brain*. **7**, 65 (2014). 25213836 10.1186/s13041-014-0065-y PMC4172865

[46] Liu, G., Weinger, J. G., Lu, Z. L., Xue, F. & Sadeghpour, S. Efficacy and safety of Mmfs-01, a synapse density enhancer, for treating cognitive impairment in older adults: A randomized, Double-Blind, Placebo-Controlled trial. *J. Alzheimers Dis.* **49**, 971–990 (2016). 26519439 10.3233/JAD-150538 PMC4927823

[47] Sun, Q., Weinger, J. G., Mao, F. & Liu, G. Regulation of structural and functional synapse density by L-Threonate through modulation of intraneuronal magnesium concentration. *Neuropharmacology* **108**, 426–439 (2016). 27178134 10.1016/j.neuropharm.2016.05.006

[48] Cao, J. et al. Combined metabolomics and machine learning algorithms to explore metabolic biomarkers for diagnosis of acute myocardial ischemia. *Int. J. Legal Med.* **137**, 169–180 (2023). 35348878 10.1007/s00414-022-02816-y

