# Supplementary material for: Metabolomic biomarkers discovery across chronic gastritis to gastric cancer progression
Source: Sci Rep. 2025 Sep 29;15:33706. doi: 10.1038/s41598-025-19005-7 (PMC12480622; doi:10.1038/s41598-025-19005-7)
Supplement: Supplementary file 1 — Supplementary Material 1 [file 41598_2025_19005_MOESM1_ESM.pdf]

Table. S1 Demographic characteristics of the control and disease groups (Sample Collection I)

Demographic	Control I	Disease I							
	(n=17)	CNAG (n=23)	Control vs. CNAG *P* value	CAG (n=23)	Control vs. CAG *P* value	GC (n=18)	Control vs. GC *P* value	All (n=64)	Control vs. All *P* value
Age (year, mean ± SD)	50.36 ±8.66	48.84 ±9.92	0.97	54 ±8.09	0.71	57.14 ±10.66	0.27	51.39 ±9.04	0.40
Gender (M/F)	10/7	12/11	0.75	10/13	0.52	13/5	0.48	35/29	0.79

Table. S2 Demographic characteristics of the control and disease groups (Sample Collection II)

Demographic	Control II	Disease II							
	(n=9)	CNAG (n=9)	Control vs. CNAG *P* value	CAG (n=9)	Control vs. CAG *P* value	GC (n=9)	Control vs. GC *P* value	All (n=27)	Control vs. All *P* value
Age (year, mean ± SD)	47 ±8.17	44.88 ±11.61	0.96	56 ±5.29	0.20	56.55 ±11.37	0.16	52.48 ±10.94	0.17
Gender (M/F)	5/4	6/3	1	3/6	0.63	6/3	1	15/12	1

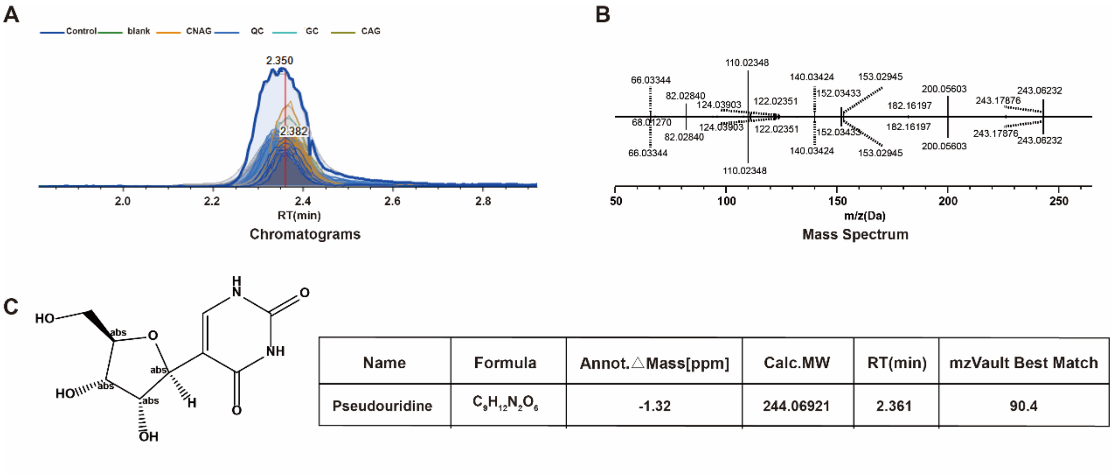

Fig. S1 Typical flowchart for metabolite identification, based on Compound Discoverer software. (A) Cumulative chromatograms of compounds for the various groups; (B) Comparison of the secondary fragments of compounds for which secondary fragments were present in the database; (C) Information regarding the compound structure, name,

and database match scores

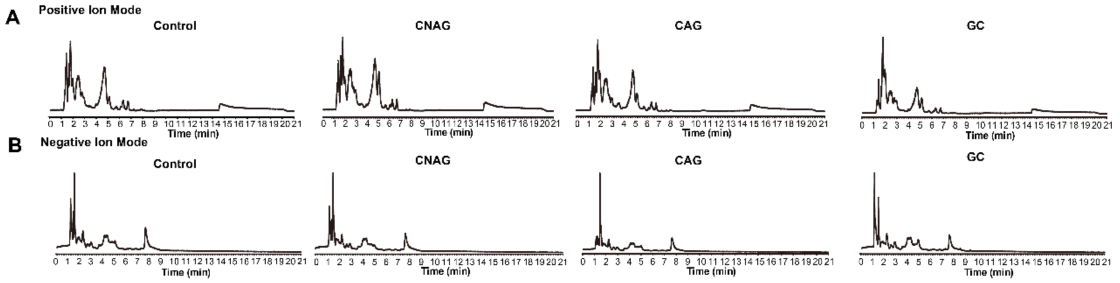

Fig. S2 Total ion chromatograms for the Control, CNAG, CAG, and GC groups. (A) Positive ion mode; (B) Negative ion mode

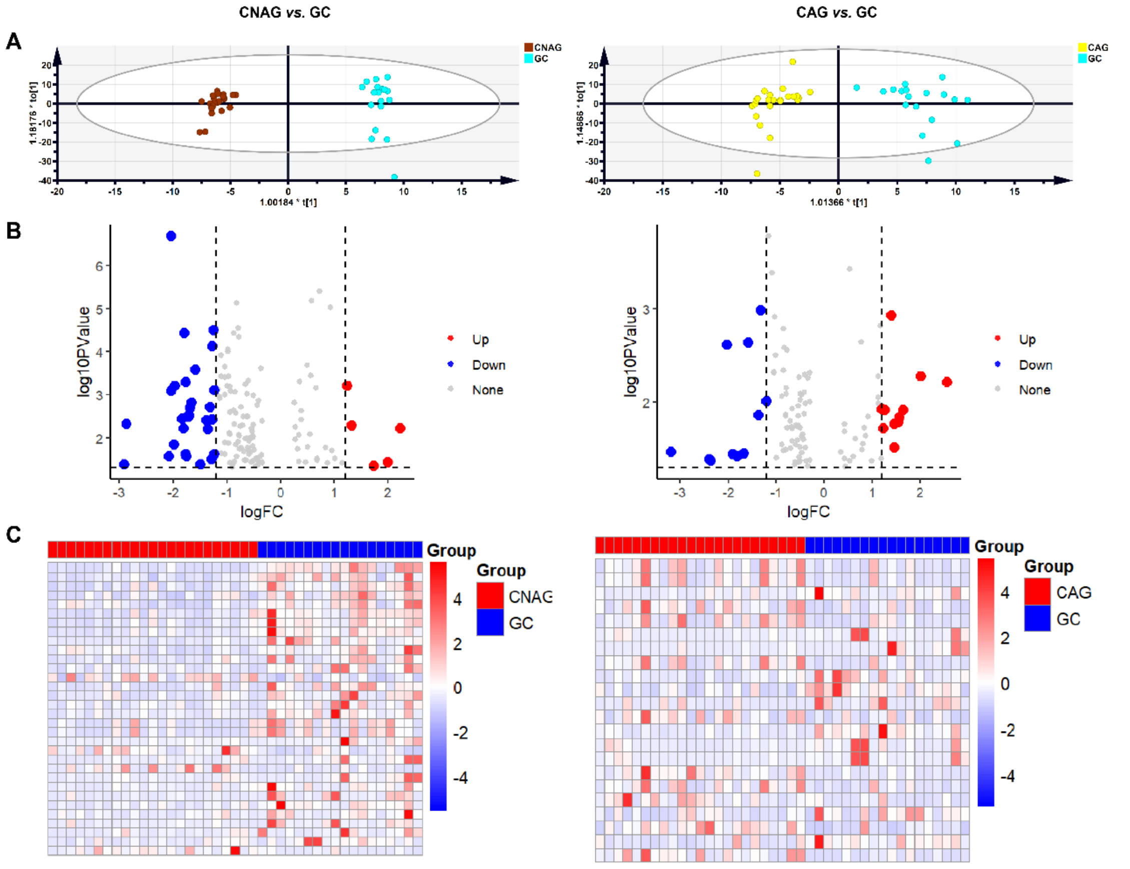

Fig. S3 Metabolites present at differing concentrations (DMs) on non-targeted metabolomics. (A) The OPLS-DA score plot for the GC vs. the CNAG and CAG groups. (B) Volcano plot of DMs for the GC vs. the CNAG and CAG groups, to identify DMs. Those with P<0.05 and |log2 FC|⩾1.2 were considered to be significant. (C) Heatmap plot of the DMs for the GC vs. the CNAG and CAG groups. CNAG: Chronic non-

atrophic gastritis; CAG: Chronic atrophic gastritis; GC: Gastric cancer.

Table. S3 Names and AUCs of the selected DMs in the different groups.

ID	Metabolites	AUC				
		Control *vs.* CNAG	Control *vs.* CAG	Control *vs.* GC	CNAG *vs.* GC	CAG *vs.* GC
(1)	[FAhydroxy(18:0)]12_13-dihydroxy-9Z-octadecenoicacid	0.571	0.511	0.611	0.474	0.579
(2)	Farylhydrazone A	0.676	0.708	0.867	0.775	0.708
(3)	O-(4,8-dimethylnonanoyl)carnitine	0.568	0.637	0.989	0.988	0.944
(4)	L-Threonic acid	0.505	0.542	0.417	0.421	0.415
(5)	Cybastacine A	0.600	0.732	0.594	0.649	0.754
(6)	Dehydroepiandrosteronesulfate(DHEAS)	0.629	0.658	0.956	0.889	0.912
(7)	Decanoylcarnitine	0.942	0.905	0.989	0.532	0.667
(8)	O-heptanoylcarnitine	0.834	0.984	0.922	0.687	0.658
(9)	PALGLY	0.968	0.889	0.961	0.579	0.570
(10)	L-Phenylalanine	0.816	0.897	0.711	0.664	0.760
(11)	[FA(20:3)]11_12-epoxy-5Z_8Z_14Z-eicosatrienoicacid	0.729	0.916	0.839	0.561	0.640
(12)	N1-(2-Ethoxyphenyl)-N2-(2-ethylphenyl)oxalamide	0.837	0.834	0.844	0.494	0.535
(13)	(2S)-1-Hydroxy-3-(pentadecanoyloxy)-2-propanyl (9Z)-9-hexadecenoate	0.668	0.689	0.922	0.860	0.813
(14)	3-(4-Methoxyphenyl)propyl hydrogen sulfate	0.737	0.674	0.922	0.819	0.825
(15)	Pseudouridine	0.863	0.958	0.700	0.719	0.895
(16)	[ST(4:0)]5beta-Cholestane-3alpha_7alpha_12alpha_26-tetrol	0.847	0.700	0.833	0.430	0.626
(17)	(14RS)-(10E,12E)-14-hydroxy-9-oxo-10,12-octadecadienoic acid	0.874	0.897	0.900	0.637	0.822
